# Epidemiology and molecular characteristics of the type VI secretion system in *Klebsiella pneumoniae* isolated from bloodstream infections

**DOI:** 10.1002/jcla.23459

**Published:** 2020-07-12

**Authors:** Mao Zhou, You Lan, Siyi Wang, Qingxia Liu, Zijuan Jian, Yanming Li, Xia Chen, Qun Yan, Wenen Liu

**Affiliations:** ^1^ Department of Clinical Laboratory Xiangya Hospital Central South University Changsha China

**Keywords:** bloodstream infections, hypervirulent, *Klebsiella pneumoniae*, T6SS, virulence factor

## Abstract

**Background:**

The type VI secretion system (T6SS) has been identified as a novel virulence factor. This study aimed to investigate the prevalence of the T6SS genes in *Klebsiella pneumoniae*‐induced bloodstream infections (BSIs). We also evaluated clinical and molecular characteristics of T6SS‐positive *K pneumoniae*.

**Methods:**

A total of 344 non‐repetitive *K. pneumoniae* bloodstream isolates and relevant clinical data were collected from January 2016 to January 2019. For all isolates, T6SS genes, capsular serotypes, and virulence genes were detected by polymerase chain reaction, and antimicrobial susceptibility was tested by VITEK® 2 Compact. MLST was being conducted for hypervirulent K. pneumoniae (HVKP).

**Results:**

69 (20.1%) were identified as T6SS‐positive *K. pneumoniae* among 344 isolates recovered from patients with BSIs. The rate of K1 capsular serotypes and ten virulence genes in T6SS‐positive strains was higher than T6SS‐negative strains (*P* = .000). The T6SS‐positive rate was significantly higher than T6SS‐negative rate among HVKP isolates. (*P* = .000). The T6SS‐positive *K. pneumoniae* isolates were significantly more susceptible to cefoperazone‐sulbactam, ampicillin‐sulbactam, cefazolin, ceftriaxone, cefotan, aztreonam, ertapenem, amikacin, gentamicin, levofloxacin, and ciprofloxacin (*P* < 0.05). More strains isolated from the community and liver abscess were T6SS‐positive *K. pneumoniae* (*P* < .05). Multivariate regression analysis indicated that community‐acquired BSIs (OR 2.986), the carriage of *wcaG* (OR 10.579), *iucA* (OR 2.441), and *p‐rmpA* (OR 7.438) virulence genes, and biliary diseases (OR 5.361) were independent risk factors for T6SS‐positive *K. pneumoniae*‐induced BSIs.

**Conclusion:**

The T6SS‐positive *K. pneumoniae* was prevalent in individuals with BSIs. T6SS‐positive *K. pneumoniae* strains seemed to be hypervirulent which revealed the potential pathogenicity of this emerging gene cluster.

## Graphical abstract

The type VI secretion system (T6SS) as a novel virulence factor. This study aimed to investigate the prevalence of the T6SS genes in Klebsiella pneumoniae‐induced bloodstream infections and the molecular characteristics of T6SS‐positive K. pneumoniae.

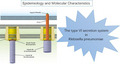

## 1 INTRODUCTION

*Klebsiella pneumoniae* is an important pathogen causing bloodstream infections (BSIs). According to the China Antimicrobial Surveillance Network (CHINET), the isolation rate of *K. pneumoniae* in blood was 15.3%, second only to *Escherichia coli*. In recent years, scholars have discovered a new type of *K. pneumoniae* called hypervirulent *K. pneumoniae* (HVKP).^1^, ^2^ Compared with classic *K. pneumoniae* (CKP), HVKP was characterized by causing severe invasive community‐acquired infections with metastatic spread in immunocompetent individuals.^3^, ^4^ Usually, hypervirulent strains were resistant to most antimicrobials.^5^ However, multidrug‐resistant HVKP strains were increasingly reported recently. The emergence of this superbug could cause severe fatal infections in both the hospital and the community.^6^, ^7^, ^8^, ^9^

Bacterial secretion systems are ubiquitous; until now, eight types of secretion systems have been described (T1SS, T2SS, T3SS, T4SS, T5SS, T6SS, T7SS, and T9SS). By secreting proteins as virulence factor, bacteria can attack other microorganisms, evade the host immune system, cause tissue damage, and invade host cells.^10^ The type VI secretion system (T6SS) is a transmembrane complex which is used to deliver effectors to hosts or target bacteria. The action process is similar to the puncture mechanism used for phage tail contraction. An effector‐loaded needle is injected into the target cell.^11^, ^12^ As an important virulence factor, T6SS plays a key role in colonization competition and infection of bacteria. Several intestinal pathogens use T6SS to antagonize symbiotic intestinal *E.coli* promoting colonization and disease progression.^13^ T6SS in *Campylobacter jejuni* has been shown to be important for adhesion and invasion of host cells in vitro.^14^ *K. pneumoniae* T6SS contributes to bacterial competition, cell invasion, type‐1 fimbriae expression, and in vivo colonization.^15^

T6SS has been identified as a novel virulence factor. There were less reports about the characteristics of BSIs caused by *K. pneumoniae* expressing T6SS genes. So, the purpose of this study was to investigate the distribution of the T6SS genes and clinical and molecular characteristics in *K. pneumoniae‐*induced BSIs.

## 2 MATERIAL AND METHODS

### 2.1 Isolates and Clinical data collection

In this study, a total of 344 non‐repetitive *K. pneumoniae* bloodstream isolates and relevant clinical data were collected from January 2016 to January 2019. Isolates were recovered from samples with positive for blood culture, after separation and cultivation, and then identified by MALDI‐TOF MS (Bruker). The distinction between community‐acquired and hospital‐acquired BSIs was determined by the time of detection of *K. pneumoniae* in blood cultures. Within 48 hours after admission was defined as community‐acquired BSIs. But over 48 hours into inpatient admission and infections correlated with the presence of medical devices was defined as hospital‐acquired BSIs.^5^, ^16^ Meanwhile, the following clinical information of the patients was collected from medical records, like age, gender, origin of bacteremia, personal history, underlying disease, and clinical outcomes.

### 2.2 Detection of T6SS genes, capsular serotypes, and virulence genes

The presence of capsular serotypes and virulence genes was detected by polymerase chain reaction (PCR) as previously described.^17^ Intracellular proliferative F family proteins (IcmF*),* valine‐glycine repeat protein (VgrG*),* and hemolysin‐coregulated protein (Hcp) were indicated to be core proteins of the T6SS.^15^ To identify the T6SS genes in *K. pneumoniae*, PCR was performed using primer pairs designed specifically for *icmF, vgrG, and hcp* in this study. Genomic DNA of *K. pneumonia* was extracted by boiling method. PCR products were electrophoresed in 1.0% agarose gel, and they were visualized using a Gel Doc ™ XR image analysis station (Bio‐Red) to judge whether the gene was positive. Strains positive for *p‐rmpA* and *iroB* and *iucA* were designated as HVKP.^18^ *icmF, vgrG, and hcp* are all positive were designated as T6SS‐positive in this study. All primers used were listed in Table 1.

**Table 1:** Primers used in this study

Primer name	DNA sequence (5′‐3′)		Amplicon size (bp)
Capsular serotypes			
K1	F: GGTGCTCTTTACATCATTGC		1283
	R: GCAATGGCCATTTGCGTTAG		
K2	F: GACCCGATATTCATACTTGACAGAG		641
	R: CCTGAAGTAAAATCGTAAATAGATGGC		
K5	F: TGGTAGTGATGCTCGCGA		741
	R: CCTGAACCCACCCCAATC		
K20	F: CGGTGCTACAGTGCATCATT		280
	R: GTTATACGATGCTCAGTCGC		
K54	F: CATTAGCTCAGTGGTTGGCT	881	
	R: GCTTGACAAACACCATAGCAG		
K57	F: CTCAGGGCTAGAAGTGTCAT	1037	
	R: CACTAACCCAGAAAGTCGAG		
Virulence genes			
*p‐rmpA*	F: CATAAGAGTATTGGTTGACAG	461	
	R: CTTGCATGAGCCATCTTTCA		
*wcaG*	F: GGTTGGKTCAGCAATCGTA	169	
	R: ACTATTCCGCCAACTTTTGC		
*allS*	F: CATTACGCACCTTTGTCAGC	764	
	R: GAATGTGTCGGCGATCAGCTT		
*iutA*	F: GGGAAAGGCTTCTCTGCCAT R: TTATTCGCCACCACGCTCTT	920	
*Aerobactin*	F: GCATAGGCGGATACGAACAT R: CACAGGGCAATTGCTTACCT	556	
*mrkD*	F: AAGCTATCGCTGTACTTCCGGCA R: GGCGTTGGCGCTCAGATAGG	340	
*Kfu*	F: GGCCTTTGTCCAGAGCTACG R: GGGTCTGGCGCAGAGTATGC	638	
*ybtS*	F: GACGGAAACAGCACGGTAAA R: GAGCATAATAAGGCGAAAGA	242	
*iucA*	F: GCATAGGCGGATACGAACAT R: CACAGGGCAATTGCTTACCT	556	
*iroB*	F: TGTGTGCTGTGGGTGAAAGC R: ATGTTCGGTGAGATTCGCCAGT	2711	
*entB*	F: GTCAACTGGGCCTTTGAGCCGTC R: TATGGGCGTAAACGCCGGTGAT	400	
T6SS genes			
*hcp*	F: TCCCGACCGATAACAACACC	242	
	R: GATGTCGTGCATCAGGGGAT		
*vgrG*	F: TGAGCGTGTTTGTGCGAAAG	259	
	R: TGACGCCCGTAATATCCTGC		
*icmF*	F: GACCGCTTACGGACAACTGA	485	
	R: CACTCAGCACCCAGTCCATT		

### 2.3 Multilocus sequence typing (MLST) and eBURST

MLST was performed for all HVKP through amplification, sequencing, and analyzing seven housekeeping genes for *K. pneumoniae*, including *gapA, infB, mdh, pgi, phoE, rpoB,* and *tonB*. Sequence types (STs) were determined according to the MLST database (https://pubmlst.org/bigsdb?db=pubmlst_mlst_seqdef). Then, analysis of genetic relationships between different STs was performed by eBURST.^19^

### 2.4 Antimicrobial susceptibility testing

All *K. pneumoniae* strains underwent antimicrobial susceptibility testing by bioMerieux VITEK® 2 Compact (bioMerieux). The bacterial suspension was added to the matching Gram‐negative bacilli susceptibility identification card for culture and identification, according to the instructions and the standard operating procedures of the instrument. A panel of 20 antimicrobial agents was tested, including cefoperazone‐sulbactam, ampicillin‐sulbactam, piperacillin‐tazobactam, cefazolin, ceftazidime, ceftriaxone, cefepime, cefotan, aztreonam, ertapenem, meropenem, imipenem, tobramycin, amikacin, gentamicin, levofloxacin, ciprofloxacin, trimethoprim‐sulfamethoxazole, furantoin, and tigecycline. Carbapenem‐resistant and extended‐spectrum β‐lactamase (ESBL)‐producing *K. pneumoniae* were also identified. The minimum inhibitory concentrations (MICs) of antimicrobial agents were interpreted according to the performance standards for antimicrobial susceptibility testing issued by the Clinical and Laboratory Standards Institute (CLSI) in 2019.^20^ *E. coli* ATCC25922, *Staphylococcus aureus* ATCC 25923, and *Pseudomonas aeruginosa* ATCC27853 were quality control strains.

### 2.5 Statistical analysis

Categorical variable analysis was used by Chi‐square test or Fisher's exact test. Student's *t* test or the Mann‐Whitney *U* test was used to analyze the measurement data. A *P* value < .05 was considered statistically significant. The virulence and clinical characteristics were summarized, and the risk factors of T6SS‐positive *K. pneumoniae*‐induced BSIs were determined by logistic regression analysis. All variables with *P* values < .1 were incorporated into a multivariate model using a backward approach. All data analysis was performed by SPSS software (version 25.0).

## 3 RESULTS

### 3.1 Distribution of T6SS genes, capsular serotypes, and virulence genes

Among 344 *K. pneumoniae* isolates recovered from patients with BSIs, 69 strains (20.1%) were positive for T6SS genes. A total of 108 isolates (31.4%) detected positive for common hypervirulent capsular types: K1, K2, K5, K20, K54, and K57. Capsular serotypes K1, K2, K5, K20, K54, and K57 comprised 38(11.1%), 36(10.5%), 4(1.2%), 3(0.9%), 3(0.9%), and 18 (5.2%) of all 344 *K. pneumoniae* strains, respectively. According to data analysis, the prevalence of K1 capsular serotype in T6SS‐positive strains was higher than T6SS‐negative strains (*P* = .000). But K20 and K54 were not detected in the T6SS‐positive strains.

As shown in Table 2, prevalence rates of eleven virulence genes were tested, including *p‐rmpA, wcaG, alls, iutA, Aerobactin, mrkD, Kfu, ybtS, iucA, iroB,* and *entB*. Except for *ybtS*, the positive rates of other virulence genes were significantly higher in T6SS‐positive strains (*P* = .000). Compared with T6SS‐negative strains, the T6SS‐positive strains had significantly higher positive rates of *p‐rmpA, wcaG, Aerobactin, Kfu, iucA,* and *iroB* (*P* < .05). As determined by positive *p‐rmpA*, *iroB,* and *iucA*, 27 strains (7.8%) were HVKP. The T6SS‐positive rate was significantly higher than T6SS‐negative rate among HVKP isolates (*P* = .000).

**Table 2:** Capsular types and virulence gene distribution of T6SS‐positive and T6SS‐negative *K. pneumoniae* bloodstream isolates

Virulence factors	All (n = 344) (%)	T6SS‐positive (n = 69) (%)	T6SS‐negative (n = 275) (%)	*P* value
Virulence gene				
*p* ***‐*** *rmpA*	79(23.0)	30(43.5)	49(17.8)	.000*
*wcaG*	39(11.3)	24(34.8)	15(5.5)	.000*
*allS*	192(55.8)	45(65.2)	147(53.5)	.079
*iutA*	99(28.8)	22(31.9)	77(28.0)	.524
*Aerobactin*	86(25.0)	30(43.5)	56(20.4)	.000*
*mrkD*	326(94.8)	67(97.1)	259(94.2)	.330
*Kfu*	86(25.0)	28(40.6)	58(21.1)	.001*
*ybtS*	192(55.8)	34(49.3)	158(57.5)	.221
*iucA*	175(50.9)	44(63.8)	131(47.6)	.017*
*iroB*	45(13.1)	20(29.0)	25(9.1)	.000*
*entB*	336(97.7)	69(100.0)	267(97.1)	.366
Capsular serotype				
K1	38(11.0)	17(24.6)	21(7.6)	.000*
K2	36(10.5)	5(7.2)	31(11.3)	.329
K5	4(1.2)	1(1.4)	3(1.1)	.804
K54	3(0.9)	0(0.0)	3(1.1)	1.000
K20	3(0.9)	0(0.0)	3(1.1)	1.000
K57	18(5.2)	5(7.2)	13(4.7)	.401
HVKP	27(7.8)	14(20.3)	13(4.7)	.000*

Abbreviations: HVKP, hypervirulent *Klebsiella pneumoniae*.

* A *P* value < .05 was considered to be statistically significant.

### 3.2 MLST and eBURST analysis

MLST analysis of 27 HVKP strains found that 13(48.1%) strains were ST23, 3 (11.1%) strains were ST268, 2 (7.4%) strains were ST25. 2 (7.4%) strains were ST375, while ST218, ST39, ST2446, ST1534, ST893, ST412, and ST65 were 1 (3.7%) strain, respectively. In Table 3, among 14 T6SS‐positive HVKP strains, ST23 was most common that reaching 11 strains (78.6%), which was much higher than T6SS‐negative HVKP (*P* = .002). However, ST268 was the common in T6SS‐negative HVKP, with 3 strains (23.1%). In ST23 HVKP strains, 9 strains (69.2%) capsular type were K1. eBURST analysis showed that ST218 and ST23 were related, and ST375 and ST25 were related. HVKP had no obvious epidemic trend during 2016 to 2019.

**Table 3:** MLST of T6SS‐positive and T6SS‐negative K. pneumoniae bloodstream isolates

Sequence types	All (n = 27)	T6SS‐positive (n = 14)	T6SS‐negative (n = 13)	*P* value
ST23	13(48.1%)	11(78.6%)	2(15.4%)	.002*
ST268	3(11.1%)	0	3(23.1%)	.098
ST25	2(7.4%)	0	2(15.4%)	.222
ST375	2(7.4%)	1(7.1%)	1(7.7%)	1.000
ST218/ST39/ST2446/ST893/ ST65	1(3.7%)	0	1(7.7%)	.481
ST1534/ST412	1(3.7%)	1(7.1%)	0	1.000

* A *P* value < .05 was considered to be statistically significant.

### 3.3 Antimicrobial resistance of T6SS‐positive and T6SS‐negative *K. pneumoniae* bloodstream isolates

Generally, all tested antimicrobial resistance of T6SS‐positive *K. pneumoniae* was lower than that of T6SS‐negative strains. Except for natural resistance to ampicillin, the highest resistance rate was *K. pneumoniae* to ampicillin‐sulbactam. Fortunately, it can be seen from Table 4 that the current resistance rate to tigecycline was low to 1.5%. The T6SS‐positive *K. pneumoniae* isolates were significantly more susceptible to cefoperazone‐sulbactam, ampicillin‐sulbactam, cefazolin, ceftriaxone, cefotan, aztreonam, ertapenem, amikacin, gentamicin, levofloxacin, and ciprofloxacin (*P* < .05). Especially, the detection rate of carbapenem‐resistant *K. pneumoniae* (CR‐KP) in the T6SS‐positive strain was also lower (*P* < .05). A summary of these results was shown in Table 4.

**Table 4:** Antimicrobial resistance of T6SS‐positive and T6SS‐negative *K pneumoniae* bloodstream isolates

Antimicrobial agent	All (n = 344) (%)	T6SS‐positive (n = 69) (%)	T6SS‐negative (n = 275) (%)	*P* value
Cefoperazone‐sulbactam	114(33.1)	15(21.7)	99(36.0)	.024*
Ampicillin‐sulbactam	199(57.8)	32(46.4)	167(60.7)	.031*
Piperacillin‐tazobactam	109(31.7)	16(23.2)	93(33.8)	.090
Cefazolin	192(55.8)	28(40.6)	164(59.6)	.004*
Ceftazidime	134(39.0)	22(31.9)	112(40.7)	.178
Ceftriaxone	173(50.3)	26(37.7)	147(53.5)	.019*
Cefepime	132(38.4)	20(29.0)	112(40.7)	.073
Cefotan	112(32.6)	15(21.7)	97(35.3)	.032*
Aztreonam	163(47.4)	23(33.3)	140(50.9)	.009*
Ertapenem	110(32.0)	15(21.7)	95(34.5)	.041*
Meropenem	102(29.7)	14(20.3)	88(32.0)	.057
Imipenem	100(29.1)	14(20.3)	86(31.3)	.072
Tobramycin	72(20.9)	9(13.0)	63(22.9)	.072
Amikacin	60(17.4)	5(7.2)	55(20.0)	.013*
Gentamicin	108(31.4)	10(14.5)	98(35.6)	.001*
Levofloxacin	118(34.3)	16(23.2)	102(37.1)	.030*
Ciprofloxacin	125(36.3)	17(24.6)	108(39.3)	.024*
Trimethoprim‐sulfamethoxazole	143(41.6)	24(34.8)	119(43.3)	.201
Furantoin	162(47.1)	27(39.1)	135(49.1)	.138
Tigecycline	5(1.5)	1(1.4)	4(1.5)	1.000
CR‐KP	110(32.0)	15(21.7)	95(34.5)	.041*
ESBL+	48(14.0)	6(8.7)	42(15.3)	.159

Abbreviations: CR‐KP, carbapenem‐resistant *Klebsiella pneumoniae;*ESBL+, producing extended‐spectrum beta‐lactamase.

* A *P* value < .05 was considered to be statistically significant.

### 3.4 Clinical characteristics

Table 5 shows the clinical characteristics of *K. pneumoniae‐*induced BSIs and T6SS‐positive and T6SS‐negative isolates. Patients of all ages with *K. pneumoniae*‐caused BSIs could be seen, while mainly were males. There was no obvious difference in age and sex between the two groups. T6SS‐positive *K. pneumoniae* was more easily acquired from the community than T6SS‐nagetive isolates. Strains isolated from liver abscess were likely to be T6SS‐positive *K. pneumoniae* (*P* < .05). It could be found from multivariate regression analysis that community‐acquired infections (OR 2.986,95% CI:1.367‐6.523), the carriage of *wcaG* (OR 10.579, 95% CI:2.589‐43.221), *iucA* (OR 2.441, 95% CI:1.085‐5.632), and *p‐rmpA* (OR 7.438, 95% CI:1.235‐44.796) virulence genes, and biliary diseases (OR 5.361,95%CI:1.428‐20.127) were independent risk factors for T6SS‐positive *K. pneumoniae*‐induced BSIs. Surprisingly, the virulence gene *ybtS* seemed to be a protective factor (OR 0.200, 95% CI: 0.083‐0.483).

**Table 5:** Clinical characteristics of T6SS‐positive and T6SS‐negative *K pneumoniae* bloodstream isolates

Characteristics	All (n = 344)	T6SS‐positive (n = 69)	T6SS‐negative (n = 275)	*P* value
Age	42.69 ± 25.83	41.96 ± 24.76	42.86 ± 26.13	.684
Gender				
Male	210(61.0%)	45(65.2%)	165(60.0%)	.427
Female	134(39.0%)	24(34.8%)	110(40.0%)	
Acquisition				
Community‐acquired	192(55.8%)	46(66.7%)	146(53.1%)	.042*
Hospital‐acquired	152(44.2%)	23(33.3%)	129(46.9%)	
Primary site				
Respiratory tract	212(61.6%)	42(60.9%)	170(61.8%)	.885
Biliary tract	15(4.4%)	1(1.4%)	14(5.1%)	.321
Intra‐abdomen	38(11.0%)	5(7.2%)	33(12.0%)	.260
Liver abscess	10(2.9%)	5(7.2%)	5(1.8%)	.016*
Brain	13(3.8%)	4(5.8%)	9(3.3%)	.326
Urinary tract	12(3.5%)	3(4.3%)	9(3.3%)	.663
Others	44(12.8%)	9(13.0%)	35(12.7%)	.944
Personal history				
Smoking history	69(20.1%)	12(17.4%)	57(20.7%)	.536
Drinking history	63(18.3%)	10(14.5%)	53(19.3%)	.359
Chemotherapy history	58(16.9%)	12(17.4%)	46(16.7%)	.895
Blood transfusion history	26(7.6%)	3(4.3%)	23(8.4%)	.318
Underlying condition				
Hypertension	83(24.1%)	17(24.6%)	66(24.0%)	.912
Cancer	59(17.2%)	12(17.4%)	47(17.1%)	.953
Diabetes mellitus	55(16.0%)	14(20.3%)	41(14.9%)	.276
Premature baby	44(12.8%)	12(17.4%)	32(11.6%)	.201
Hematological diseases	41(11.9%)	7(10.1%)	34(12.4%)	.611
Biliary tract disease	36(10.5%)	11(15.9%)	25(9.1%)	.096
Pulmonary infection	33(9.6%)	6(8.7%)	27(9.8%)	.777
Acute severe pancreatitis	28(8.1%)	6(8.7%)	22(8.0%)	.850
Liver cirrhosis	13(3.8%)	3(4.3%)	10(3.6%)	.729
Multiple bacterial infections	66(19.2%)	11(15.9%)	55(20.0%)	.444
Septic shock	85(24.7%)	13(18.8%)	72(26.2%)	.206
Death in hospital	19(5.5%)	6(8.7%)	13(4.7%)	.197

* A *P* value < .05 was considered to be statistically significant.

## 4 DISCUSSION

This retrospective study analyzed the prevalence, and molecular and clinical characteristics of 344 patients with *K. pneumoniae*‐induced BSIs from January 2016 to January 2019. It was the first study that focusing on the new virulence factor T6SS in *K. pneumoniae* bloodstream isolates.

Hypermucoviscosity and strong iron acquisition systems were important characteristic of HVKP. ^21^, ^22^ Hence, like the majority researches, strains positive for *p‐rmpA*, *iroB,* and *iucA* were considered as HVKP in this study, and the results demonstrated that 27(7.8%) strains were HVKP.^18^ The prevalence rate of *K. pneumoniae‐*induced BSIs was 7.8%, similar to the study conducted in Spain, but much lower than previous studies conducted in China (24.5% or 21.6%).^23^, ^24^, ^25^ The inconsistent definition of HVKP may be the cause of this phenomenon. “String test” was widely used to identify HVKP in most previous studies, while it was confirmed that it did not distinguish HVKP from CKP. A clear and unified identification of HVKP was urgently needed.

Currently, T6SS has been identified as a virulence factor, which can inject enzymes, toxins, or other proteins into competing bacteria or host cells, and secrete proteins as virulence factors.^26^ As an important core protein of the T6SS, VgrG forms a cell‐puncturing tip and Hcp forms a tail‐tube structure for transport effector proteins.^27^ VgrG was not only a directly interact device, but also a secreted protein of T6SS, which exerted virulent infections. ^28^, ^29^ When VgrG was separated from the Hcp tube, the secreted proteins of T6SS were also released into the host cell through the Hcp tube.^30^ IcmF were the conservatively integrated inner membrane proteins of T6SS and responsible for delivering effector proteins to target cells.^31^ The sequencing results of *K. pneumoniae* indicated that NTUH‐K2044 *K. pneumoniae* had two gene Loci, Locus I contained protein‐encoding genes secreted by *hcp, vgrG,* and *icmF,* Locus III contained *vgrG* and *icmF* genes. So, in our study, strains positive for *icmF, vgrG, and hcp* were designated as T6SS‐positive. Based on this standard, our study indicated that the frequency of T6SS genes among *K. pneumoniae* bloodstream isolates was 20.1%, which was lower than *K. pneumoniae* isolated from pyogenic liver abscess (PLA) (88.1%) and the intestinal (41.5%).^15^

As a key virulence factor, K1 and K2 were most associated with hypervirulent of all capsular serotypes among *K. pneumoniae*.^32^ We used PCR to test six common high‐virulence‐associated capsular serotypes. K1 was most frequently in *K. pneumoniae* bloodstream isolates, followed by K2. Analysis revealed that detection rate of K1 in T6SS‐positive strains was significantly higher than T6SS‐negative strains. In addition, there was a study demonstrated that T6SS genes contributes to the development of meningitis caused by K1 *E. coli*.^33^ Taken together, T6SS‐positive *K. pneumoniae* strain seems to have a strong virulence potential.

The virulence of the T6SS‐positive *K. pneumoniae* strains was further supported by this study that the positive rates of the virulence genes except for *ybts* were higher in T6SS‐positive than T6SS‐negative strains. More importantly, *p‐rmpA, wcaG, Aerobactin, Kfu,* and *iucA* were related to hypervirulent, hypermucoviscosity phenotype, and iron acquisition.^34^, ^35^, ^36^, ^37^ Moreover, the rate of T6SS‐positive strains was significantly higher than T6SS‐negative strains among HVKP. Whole genome sequencing declared that genes coding for iron uptake systems are encoded in adjacencies of T6SS, suggesting that T6SS might play a role in iron import.^38^ As is known to all, iron acquisition is a vital part of HVKP. These findings further supported the view that T6SS‐positive strains may have a relationship with hypervirulence.

Similar to other studies, the most prevalent ST in HVKP isolates was ST23. ^5^ Among the 13 ST23 HVKP isolates, 11(78.6%) were T6SS‐positive and only 2 were T6SS‐negetive. In ST23 strains, 9(69.2%) capsular type were K1. ST23 seemed to be related to K1, which had been shown by many studies.^39^, ^40^, ^41^ Several researches proved that ST23 was closely related to *K. pneumoniae* virulence.^34^, ^42^, ^43^ Complexing capsular serotypes, virulence genes, and hypervirulence‐associated ST23, there was an evidence that T6SS‐positive *K. pneumoniae* strains was hypervirulent.

The antimicrobial resistance rates of T6SS‐positive *K. pneumoniae* strains in this study were lower than T6SS‐negetive. But carbapenem‐resistant, tigecycline‐resistant, and ESBL‐producing *K. pneumoniae* still existed. As we know, hypervirulent strains were usually sensitive to antimicrobials. Also, HVKP were difficult to obtain or lose resistance‐associated plasmids, and capsules can also affect the horizontal spread of resistant genes.^44^ However, the studies about ESBLs‐producing, carbapenemase‐resistant, even NDM‐1 HVKP strains have been reported increasingly in recent years.^6^, ^7^, ^8^, ^9^, ^45^ Once hypervirulence and high resistance characteristics are combined, it will undoubtedly become a great threat to public health.

HVKP was characterized by causing severe and spreadable community‐acquired infections like liver abscess in young healthy people.^46^, ^47^ Analysis of clinical characteristics showed that T6SS‐positive *K. pneumoniae* was more easily acquired from the community, which was also a manifestation of hypervirulent. More T6SS‐positive *K. pneumoniae* were isolated from patients with liver abscess. There was a study about *K. pneumoniae* isolated from PLA claimed that T6SS genes aid interspecies and intraspecies antibacterial competitiveness, mediate in the transcriptional expression of type‐1 fimbriae, and promote the occurrence of liver abscesses.^15^ Besides, it was worth noting that biliary disease seemed to be related to T6SS‐positive *K. pneumoniae*. However, biliary tract infections were mostly caused by CKP in previous studies.^48^ The coordinated regulation of T6SS and the bile efflux transporter ensuring *C. jejuni* survival during exposure to the upper range of physiological concentrations of deoxycholicacid.^14^ The adaptive mechanism may also exist in T6SS‐positive *K. pneumoniae, and* relevant researches are required to corroborate the association between T6SS‐positive *K. pneumoniae* and biliary disease.

In conclusion, the prevalence of T6SS genes is high among *K. pneumoniae* BSIs. T6SS‐positive strains exhibit hypervirulent properties and potential pathogenicity. Community‐acquired infections, the carriage of *wcaG, iucA,* and *p‐rmpA* virulence genes, and biliary diseases were independent risk factors, of T6SS‐positive *K. pneumoniae‐*induced BSIs. This study introduced the molecular and clinical characteristics of T6SS‐positive *K. pneumoniae* isolated from BSIs which make clinicians aware of the importance in epidemiologic surveillance of this gene cluster. Furthermore, it can also contribute to the in‐depth study about virulence mechanism of *K. pneumoniae*.

## CONFLICTS OF INTEREST

The author reports no conflicts of interest in this work.

## ETHICAL APPROVAL

The study was approved by the Ethics Committee of Xiangya Hospital, Central South University. No informed consent was taken because this study was retrospective, and it did not cause additional medical procedure.
